# Negative-pressure wound therapy for fixing full-thickness skin graft on the thumb

**DOI:** 10.1016/j.jpra.2018.08.001

**Published:** 2018-08-23

**Authors:** Yosuke Niimi, Hiroshi Ito, Hiroyuki Sakurai

**Affiliations:** a Department of Plastic and Reconstructive Surgery, Tokyo Women's Medical University; Shinjuku-ku, Tokyo, Japan; b Department of Surgery, Plastic and Reconstructive Surgery Division, University of Miyazaki Faculty of Medicine, Miyazaki-shi, Miyazaki, Japan

**Keywords:** NPWT, Finger, Hand, Reconstruction, FTSG, PICO

## Abstract

Negative-pressure wound therapy (NPWT) is used not only for preparing wound bed but also for fixing the skin graft. However, there is no report describing the use of NPWT after full-thickness skin graft (FTSG) on the digits. Because NPWT causes a leak easily, especially in the digital region, this study reported a new technique using an NPWT device without leaking and the efficacy of NPWT after FTSG.

A 51 year old male had 35 × 15 mm skin ulcer on the palmar side of the right thumb after microsurgical replantation surgery for treating a finger amputation. FTSG was performed as day surgery, and the grafted site was covered with an NPWT device. NPWT was fixed by a “sandwich technique” with two sheets covering both the dorsal and pulp sides of the thumb. The graft was perfectly planted, and the morphology was favorable at 12 months after skin grafting. FTSG with NPWT was considered as a cost efficient and effective treatment for the digit.

## Introduction

Full-thickness skin graft (FTSG) on digits is commonly required for treating various injuries, skin defect after reconstructive surgery, and congenital anomalies from the viewpoints of aesthetic and functional outcome. However, (1) its lower graft take rate than split-thickness skin graft (STSG), (2) the high medical cost, and (3) the contracture of the finger joint following the use of splint and/or pinning to be kept on the grafted site are still challenging problems. Herein, we reported the efficacy of negative-pressure wound therapy (NPWT) to the digit sites, which are known to be difficult to treat. This case study described (1) a technique for applying NPWT and (2) the efficacy of NPWT after FTSG on the digit site.

## Case presentation

**Figure 1:**
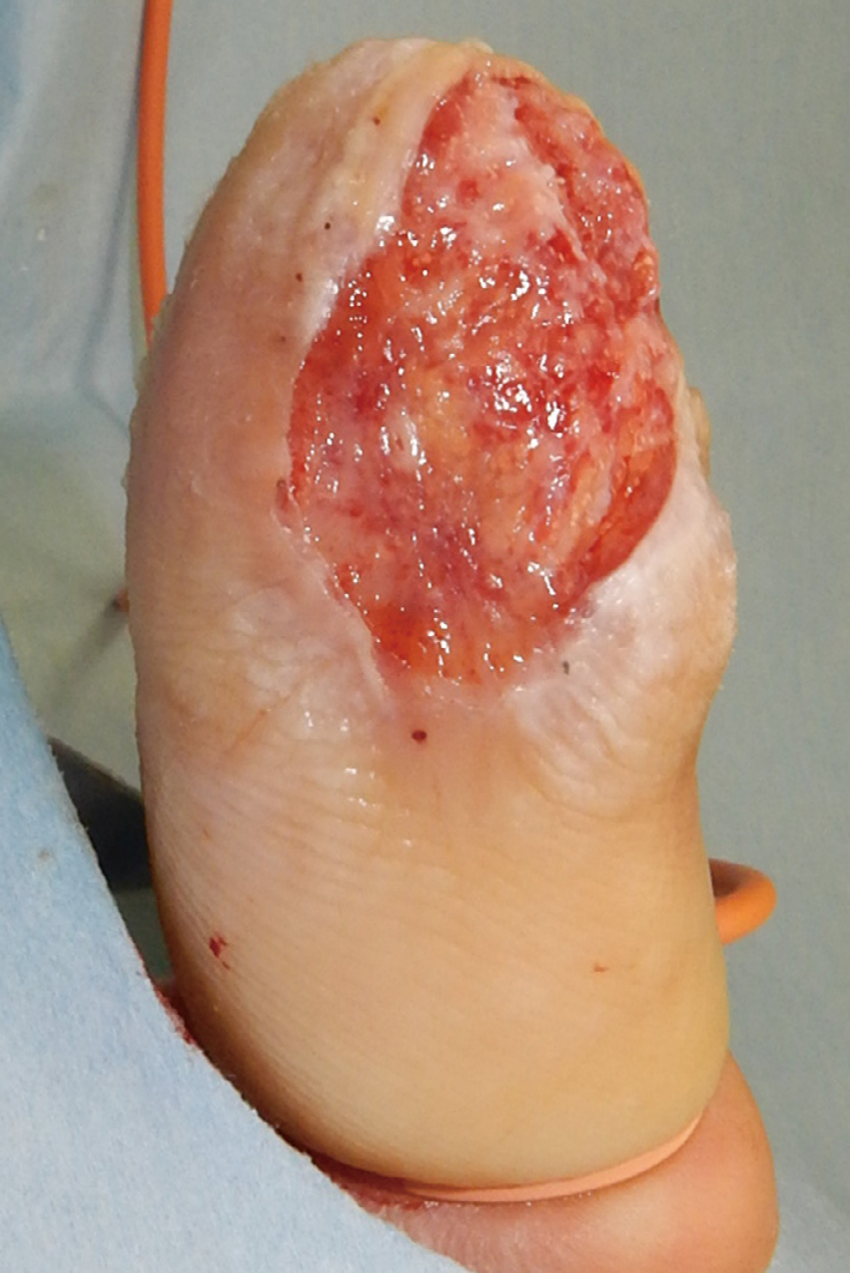
Preoperative finding on the right thumb of a 51 year old male. A 35 × 15 mm-skin ulcer on the palmar side was noticed.

**Figure 2:**
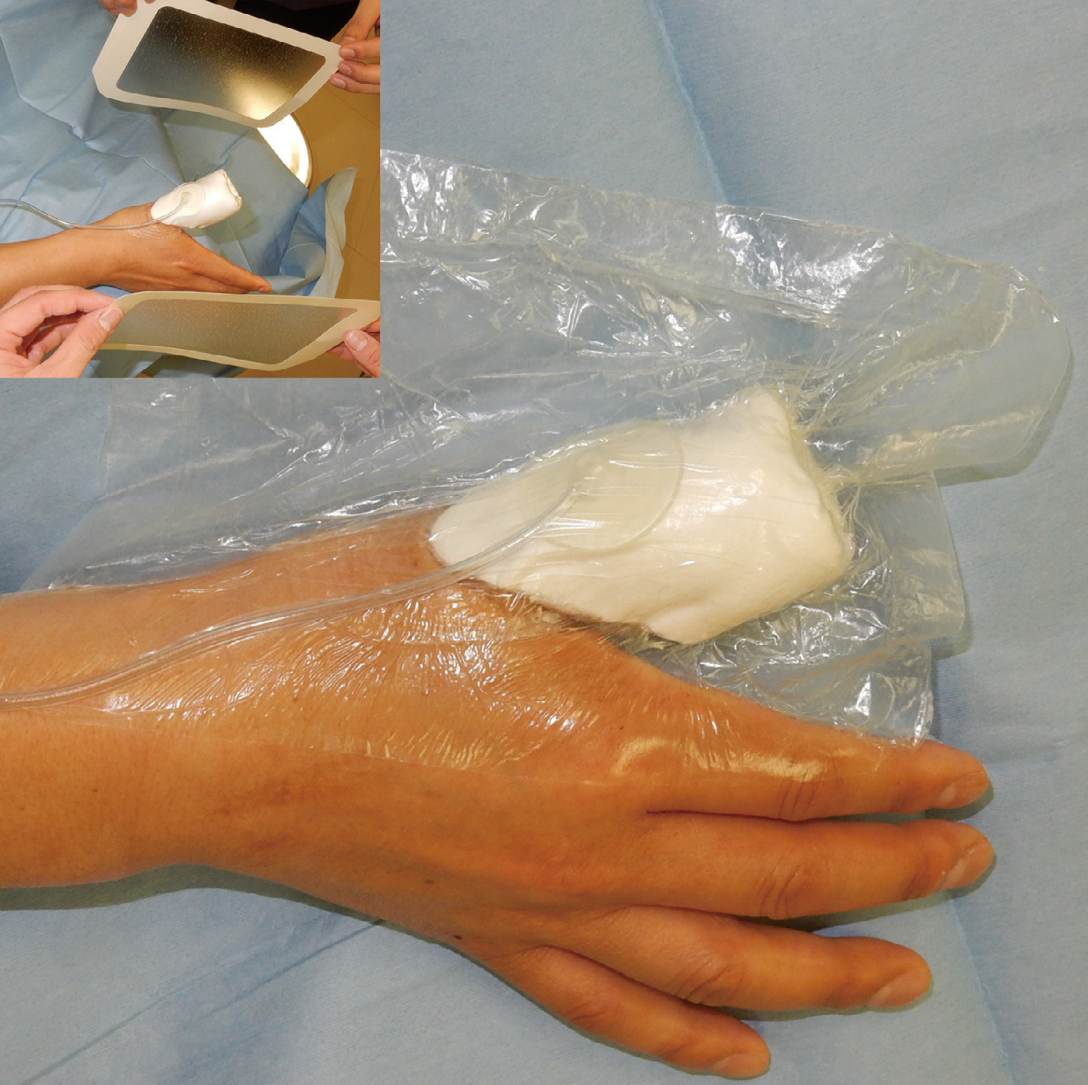
Application of a single-use negative-pressure wound therapy system to fix full-thickness skin graft (FTSG). (Inset) After dressing was applied to the FTSG site and the aspiration port was made onto the dorsal side of digit, two polyurethane film dressings were allowed to sandwich both the dorsal and palmar sides of the thumb. After applying the sheets, air between the sheets was aspirated and no air leakage was confirmed.

**Figure 3:**
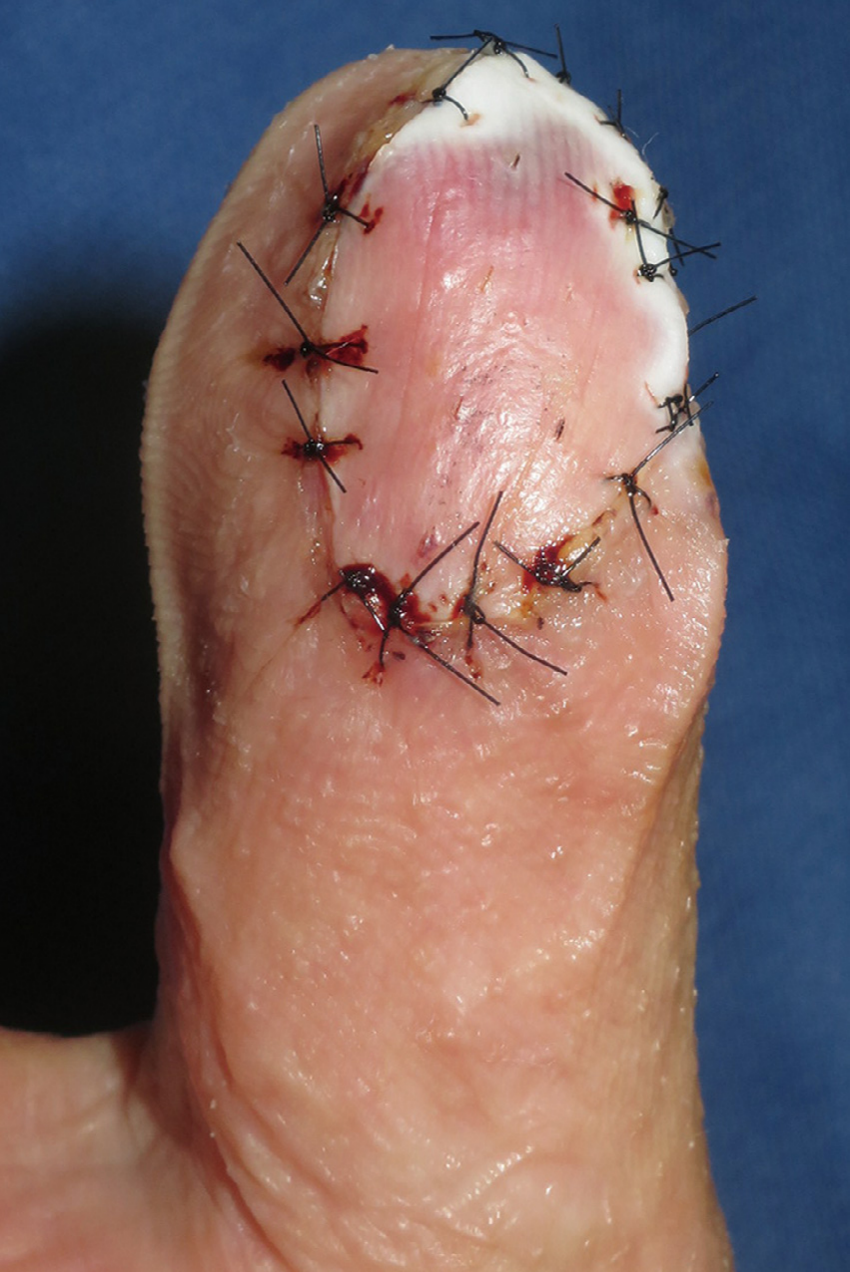
Situation of the thumb after removing the device at 5 days after surgery. Full-thickness skin graft was observed to be perfectly planted to the thumb without any complications.

**Figure 4:**
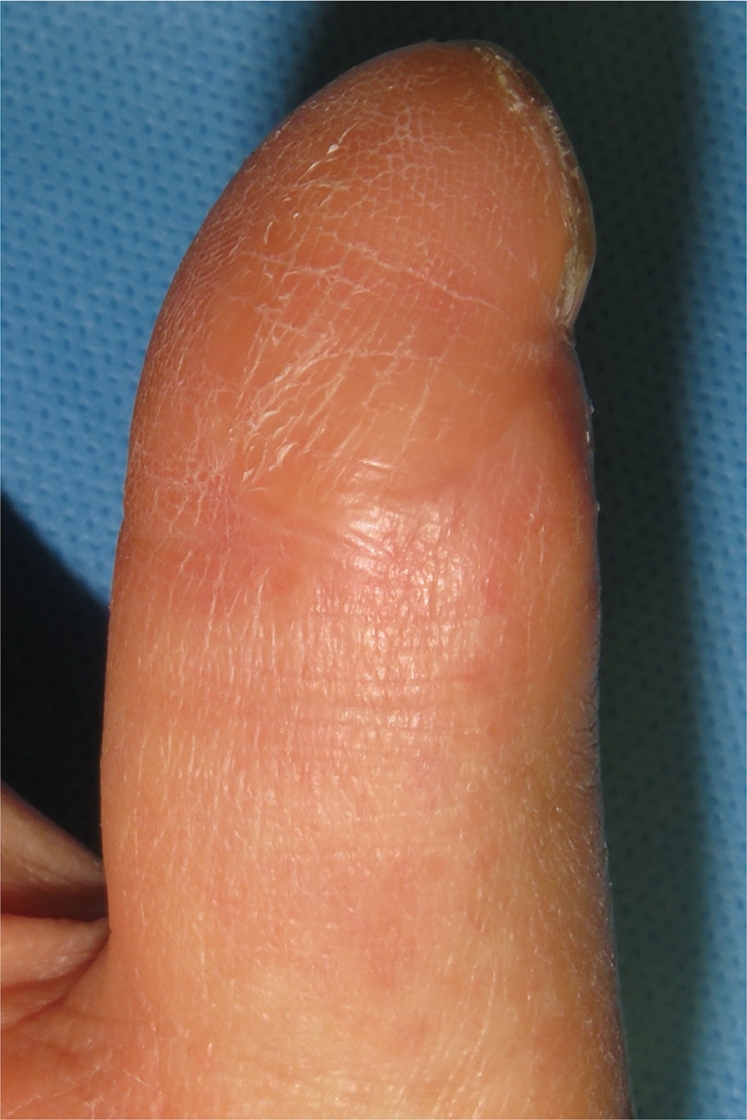
At 12 months after skin grafting, the morphology and skin texture were favorable without skin contracture.

The patient was a 51 year old male with no medical history. After being injured in a door, his right thumb sustained an Ishikawa subzone II amputation with crush injury. Although microsurgical replantation was performed, the replanted finger found to be partial necrosis from palmar to radial side of the thumb. Eventually, a 35 × 15 mm skin ulcer was noticed (Figure 1). FTSG harvested from the right medial foot was transplanted at 1 month after microsurgical replantation surgery. After the grafted skin was sutured with 5-0 nylon, a single-use NPWT system (PICO, Smith & Nephew, London, UK) was applied to fix the skin graft. After blood was removed from around the grafted site, the dressing was applied to the whole thumb including FTSG site, and the aspiration port was made onto the dorsal side. Two polyurethane film-dressing sheets (Tegaderm) (3M, St. Paul, MN, USA) were allowed to sandwich both dorsal and palmar sides of thumb (Inset in Figure 2). In this case, the first interdigital space and thenar part were also sandwiched with the sheets to prevent air leak from the site area. Then air between the sheets was aspirated (Figure 2). The sandwiching sheets gave an NPWT condition at a single preset pressure of −80 mmHg. No bleeding and air leakage were confirmed after applying vacuum. Because the patient was able to fully mobilize the hand within the dressing, he could start to rehabilitate interphalangeal joint and metacarpophalangeal joint immediately after the surgery to prevent finger contracture. During that period, no complications such as air leakage and pain at the grafted sites were found. Because the dressing has a siliconized surface, it was easy to remove from the underlying skin graft and painless for the patient at day 5. The FTSG was taken fully, and no complications such as wound infection, pressure pores, blisters, and ischemia of the thumb were observed in NPWT applied area including the wound (Figure 3). Thereafter, the wound was treated conservatively with vaseline ointment. At 12 months after skin grafting, the morphology and skin texture were favorable without complications such as pigmentation and contracture (Figure 4).

## Discussion

This study suggested NPWT was an effective dressing for FTSG, which is well known to give a lower graft take rate than STSG.^1^ The mechanisms of NPWT for fixing the skin graft are (1) the removals of exudate and bacteria, (2) improvement in tissue oxygenation,^2^ (3) equal pressure on irregular wound or highly mobile area,^3^ and (4) the immobilization of grafted site without splint.^3^ Although the single preset of −80 mmHg NPWT device was used in this study, no complaints such as hematoma, seroma, and pressure pore were noticed immediately after treatment, and a good grafting rate and a favorable esthetic outcome without contracture were found after long-term observation.

Although some reports show that one of the benefits of NPWT for skin graft is short hospital stay,^2^ the authors also think that NPWT was considered as a cost-effective treatment because the patients could be treated by day surgery without admission.

Applying NPWT for the hand is currently known to be difficult. There are some reports on preventing air leak and promoting rehabilitation.^4^ The sheet sandwiching technique in this report had the following benefits: (1) It was easy to use and time saving to perform, (2) it was easy to find leak sites, and (3) the dressing was able to be cut according to the shape of wound area. Consequently, this technique allowed the patient to start rehabilitation in the early phase without air leakage. This method expects to be used in various sites such as the hand, foot, and auricle. Although NPWT is known to be safe for peripheral artery disease, the effects of NPWT on the site of vascular anastomosis are unclear.^5^ Skin graft at an anastomosis site should be investigated carefully.

Although as a limitation, this study showed only one case, the authors will accumulate cases and evaluate the grafting rate and medical cost with or without NPWT.

FTSG with NPWT was considered as a cost efficient and effective treatment for digits.

## References

[1] Prasetyono TO, Sadikin PM, Saputra DK (2015). The use of split-thickness versus full-thickness skin graft to resurface volar aspect of pediatric burned hands: a systematic review. Burns.

[2] Moisidis E, Heath T, Boorer C, Ho K, Deva AK (2004). A prospective, blinded, randomized, controlled clinical trial of topical negative pressure use in skin grafting. Plast Reconstr Surg.

[3] Hoeller M, Schintler MV, Pfurtscheller K (2014). A retrospective analysis of securing autologous split-thickness skin grafts with negative pressure wound therapy in paediatric burn patients. Burns.

[4] Kamolz LP, Lumenta DB (2013). Topical negative pressure therapy for skin graft fixation in hand and feet defects: a method for quick and easy dressing application–the “sterile glove technique”. Burns.

[5] Niimi Y, Mori S, Takeuchi M (2017). A new procedure for wrapped-negative pressure wound therapy for congestion after arterialized venous flap surgery. Clin Med Insights Case Rep.

